# Editorial: Inflammation and chronic disease

**DOI:** 10.3389/fmed.2024.1434533

**Published:** 2024-07-02

**Authors:** Frank A. Orlando, Arch G. Mainous

**Affiliations:** ^1^Department of Community Health and Family Medicine, University of Florida, Gainesville, FL, United States; ^2^Department of Health Services Research, Management and Policy, University of Florida, Gainesville, FL, United States

**Keywords:** inflammation, chronic disease, cardiovascular, cancer, CRP, cardiometabolic, social determinants of health, clonal hematopoiesis

## Introduction

**Figure 1:**
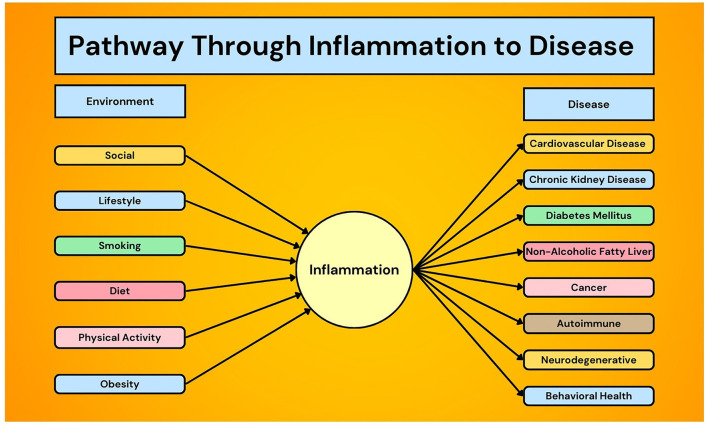
Unifying theory of inflammation and chronic disease prevention.

Inflammation is directly associated with the morbidity and mortality of a diverse number of chronic health conditions including cardiovascular disease (1–3), chronic kidney disease (4), diabetes mellitus (5, 6), non-alcoholic fatty liver disease (7), cancer (8, 9), autoimmune diseases (10, 11), and neurodegenerative (12) and behavioral health disorders (13) (Figure 1). Not only is inflammation the byproduct of chronic disease, it also has a mechanistic role in the underlying etiology and pharmacoprevention of diseases such as atherosclerosis (14). For example, some of the most common mutations in age-related, clonal hematopoiesis of indeterminate potential (CHIP) increase the expression of inflammatory genes, potentially explaining why CHIP is associated with almost twice the risk of coronary artery disease (15–17).

Various acute and chronic factors can modulate inflammation, including infection, the social and physical environments (18), lifestyle (19–21), diet (22, 23), and physical activity (24–27) (Figure 1). A PubMed search of “chronic inflammation” leads to over 170,000 results, and stalwart medical institutions propose diets targeting chronic inflammation (28, 29). Moreover, translating such knowledge to disease therapy has improved outcomes, such as using exercise to reduce inflammation in patients with depression (30), COPD (31), and frailty (32). Despite this, there is a lack of anti-inflammatory guidelines to prevent and treat chronic disease from bench to practice, and a deeper understanding of the complex relationship between inflammation and chronic disease development and progression is needed.

## Heart disease and cardiometabolic disease

A body of experimental evidence demonstrates how interferons (IFNs) and IFN-related pathways play important roles both in the inflammation commonly associated with heart disease pathogenesis as well as in the protection against heart disease (Tran et al.). While it is unlikely that measuring a single plasma IFN will be of prognostic significance in managing heart disease, immense advances in single cell technologies are helping elucidate the molecular mechanism of heart disease. Therapeutic, immunosuppressive strategies to reduce IFN or IFN-related pathway signaling come with an increased malignancy and infection risk, and targeting downstream pathways, such as cyclic GMP-AMP synthase-stimulator of interferon genes signaling, may theoretically overcome these side effects by allowing other immune defense pathways to remain intact (Tran et al.). When the advanced lung cancer inflammation index (ALI) was used to assess inflammation in hypertensive patients, it determined that lower levels of inflammation (i.e., higher ALI) were associated with reduced risk of cardiovascular death (Tu et al.). However, because of the way ALI uses body mass index (BMI) in its equation and how high BMI is an established risk factor for cardiovascular death, it is recommended that ALI not be used as a prognostic marker for cardiovascular death in hypertensive patients with BMI ≥35.5 kg/m^2^.

US adults with undiagnosed cardiometabolic disease have a higher risk of elevated HsCRP (Mainous, Sharma et al.). Furthermore, risk of the metabolic disorder nonalcoholic fatty liver disease (NAFLD) was linearly associated with the inflammation-related biomarkers SII, neutrophil-to-lymphocyte ratio (NLR), and lymphocyte-to-monocyte ratio (LMR) and non-linearly associated with platelet-to-lymphocyte ratio (PLR) when natural logarithm (ln) transformed (Liu et al.). These results further elucidate inflammation's clinical significance in NAFLD may assist with ongoing research to improve diagnosis and treatment options.

## Cancer and immunoinflammatory dermatoses

Levels of vascular adhesion protein-1 (VAP-1), a dual-function glycoprotein with an important role in inflammation and tumor progression, was associated with an increased 12-year risk of cancer incidence, cancer mortality, and all-cause mortality in a Taiwanese population, a predictive performance that was better than smoking (Chen et al.). Two inflammation-related biomarkers, systemic immune-inflammation index (SII) and product of platelet count and neutrophil count (PPN), were independent risk factors for kidney cancer incidence and may aid in the development of targeted screening strategies for at-risk patients (He et al.). Inflammasomes, immune-functional protein multimers that are closely linked to the host defense mechanism and can activate various inflammatory signaling pathways closely associated with malignancies, have become a novel target of more than 50 natural extracts and synthetic small molecule agents as prospective therapies for common cancers (Gu et al.). Another novel inflammatory target for cancer therapeutics are neutrophil extracellular traps (NETs), web-like structures containing DNA and released from the nucleus or mitochondria (Zhong et al.). NETs, important structures in innate immunity and the progression of inflammatory diseases, are being investigated for their role in potentially treating multiple cancers, especially metastatic cancer.

Circulating inflammatory cytokines' role in the development of immunoinflammatory dermatoses offers new prevention and therapeutic targets. In a Mendelian randomization study, both IL-4 and IL-1RA may have inhibitory functions in atopic dermatitis pathogenesis (Li et al.). Conversely, IL-4 and SCGF-b may have promoting functions in the pathogenesis of vitiligo and psoriasis, respectively.

## Social environment, diet, and physical activity

A considerable proportion of US adults have elevated inflammation as measured by highly sensitive C-reactive protein (HsCRP), especially minorities and individuals with low socioeconomic status (Mainous, Orlando et al.). While either inflammation or poverty alone each confer about a 50% increased risk in all-cause mortality in US adults aged 40 and older, individuals with both inflammation and poverty have a 127% increased heart disease mortality risk and a 196% increased cancer mortality risk, revealing that the combined effect of inflammation and poverty on mortality is synergistic in this population (Mainous, Orlando et al.). Therefore, both systemic inflammation and poverty could become a focus of primary care for preventing disease and monitoring its progression.

Low-grade chronic inflammation can be initiated and aggravated by specific key dietary factors, particularly sugars and mixed processed foods, the consumption of which has significantly increased over the past 30 years (Ma et al.). The negative impact that a high-sugar diet has on certain autoimmune conditions, such as rheumatoid arthritis, multiple sclerosis, psoriasis, inflammatory bowel disease, has been mechanistically linked to its pro-inflammatory effects (Ma et al.). For example, obesity has been strongly associated with low-grade chronic inflammation, but in the case of psoriasis, new research suggests that dietary sugars and fats mediate the inflammatory stimulation of psoriasis rather than obesity itself (Ma et al.). Similarly, physical activity influences inflammation with connections to disease severity. For example, physical activity following surgical resection for colon cancer is associated with a significantly increased disease-free survival, and inflammation has been hypothesized to be the linking factor (Brown et al.). Even though aerobic exercise was not associated with dose-response reductions in HsCRP or IL-6 in a randomized, dose-response trial of 39 stage I-III colon cancer survivors, cancer stage modified the association (Brown et al.). Specifically, exercise was not associated with inflammatory biomarkers in stage I-II disease, and 300 min/week of moderate-intensity aerobic exercise (high-dose) was not associated with inflammation in stage III disease, but 150 min/week of moderate-intensity aerobic exercise (low-dose) did reduce HsCRP and IL-6 in stage III disease (Brown et al.). However, the biological reason why cancer stage modifies the association between exercise dose and inflammatory biomarker levels remains unclear and is being prospectively studied in an ongoing randomized trial of exercise in colorectal cancer survivors (NCT03975491).

## Conclusion

Inflammation has an established connection with the etiology and progression of numerous major chronic diseases, but no specific guidelines exist for clinicians to use inflammatory markers to guide prevention, diagnosis, or treatment. Equally as important, the opportunity to discover a breakthrough treatment for such common chronic diseases may be right at our fingertips with the mechanistic knowledge of inflammation's role in disease pathogenesis. Future large, prospective clinical trials are needed to further elucidate the findings of mechanistic and observational trials and translate them to the bedside.

## Author contributions

FO: Conceptualization, Investigation, Writing – original draft. AM: Conceptualization, Supervision, Writing – review & editing.
